# Isatuximab plus pomalidomide and dexamethasone in relapsed/refractory multiple myeloma patients with renal impairment: ICARIA-MM subgroup analysis

**DOI:** 10.1038/s41375-020-0868-z

**Published:** 2020-05-23

**Authors:** Meletios A. Dimopoulos, Xavier Leleu, Philippe Moreau, Paul G. Richardson, Anna Marina Liberati, Simon J. Harrison, H. Miles Prince, Enrique M. Ocio, Sylvie Assadourian, Frank Campana, Laure Malinge, Dorothée Sémiond, Helgi van de Velde, Kwee Yong

**Affiliations:** 1 Department of Clinical Therapeutics, School of Medicine, National and Kapodistrian University of Athens School of Medicine, Athens, Greece; 2 Department of Hematology, Centre Hospitalier Universitaire, Université de Poitiers, Poitiers, France; 3 University of Nantes, Nantes, France; 4 Medical Oncology, Dana-Farber Cancer Institute, Boston, MA USA; 5 Oncology-Hematology, Santa Maria Hospital, University of Perugia, Terni, Italy; 6 Clinical Haematology, Peter MacCallum Cancer Centre and Royal Melbourne Hospital; Sir Peter MacCallum Department of Oncology, University of Melbourne, Melbourne, VIC Australia; 7 Epworth Healthcare, University of Melbourne, Melbourne, VIC Australia; 8 Hospital Universitario Marqués de Valdecilla (IDIVAL), Universidad de Cantabria, Santander, Spain; 9 Sanofi R&D, Paris, France; 10 Sanofi-Genzyme Oncology, Cambridge, MA USA; 11 Aixial (for Sanofi), Boulogne-Billancourt, France; 12 Department of Haematology, University College London Hospitals NHS Foundation Trust, London, UK

**Keywords:** Immunotherapy, Cancer immunotherapy, Myeloma

## Abstract

The randomized, phase 3 ICARIA-MM study investigated isatuximab (Isa) with pomalidomide and dexamethasone (Pd) versus Pd in patients with relapsed/refractory multiple myeloma and ≥2 prior lines. This prespecified subgroup analysis examined efficacy in patients with renal impairment (RI; estimated glomerular filtration rate <60 mL/min/1.73 m²). Isa 10 mg/kg was given intravenously once weekly in cycle 1, and every 2 weeks in subsequent 28-day cycles. Patients received standard doses of Pd. Median progression-free survival (PFS) for patients with RI was 9.5 months with Isa-Pd (*n* = 55) and 3.7 months with Pd (*n* = 49; hazard ratio [HR] 0.50; 95% confidence interval [CI], 0.30–0.85). Without RI, median PFS was 12.7 months with Isa-Pd (*n* = 87) and 7.9 months with Pd (*n* = 96; HR 0.58; 95% CI, 0.38–0.88). The overall response rate (ORR) with and without RI was higher with Isa-Pd (56 and 68%) than Pd (25 and 43%). Complete renal response rates were 71.9% (23/32) with Isa-Pd and 38.1% (8/21) with Pd; these lasted ≥60 days in 31.3% (10/32) and 19.0% (4/21) of patients, respectively. Isa pharmacokinetics were comparable between the subgroups, suggesting no need for dose adjustment in patients with RI. In summary, the addition of Isa to Pd improved PFS, ORR and renal response rates.

## Introduction

Renal impairment (RI) affects up to 50% of patients with multiple myeloma (MM) [1]. In most patients, this is due to the accumulation and precipitation of immunoglobulin free light chains in the distal tubules, resulting in tubule obstruction and cast nephropathy [1]. RI is an independent predictor of adverse survival outcomes for myeloma patients [2, 3] and the median survival of patients with RI is approximately half that of patients without RI [2]. Anti-myeloma treatments that can also improve renal function are urgently required.

Patients with RI are often excluded from or underrepresented in clinical trials [4, 5], and historically, the criteria for renal improvement have been inconsistent between studies. Recent International Myeloma Working Group (IMWG) recommendations defined complete renal response as an increase in estimated glomerular filtration rate (eGFR) from <50 mL/min/1.73 m² at baseline to ≥60 mL/min/1.73 m² (eGFR ≥60; no RI) in at least one post-baseline assessment [6]. Sustained complete renal responses are complete responses lasting at least two months [7].

Based on its anti-myeloma capacity, and a route of metabolism that is unaffected by renal function, bortezomib-based regimens are currently recommended by IMWG as the first choice of treatment for patients with MM-related RI [6]. Bortezomib-based triplet therapy has resulted in renal recovery in newly diagnosed patients with RI [8], including when used in combination with melphalan and prednisone, or doxorubicin and dexamethasone [9, 10]. The phase 3 MMY-3021 study demonstrated that similar results for renal response are achieved with subcutaneous administration of bortezomib [11].

Carfilzomib has also been shown to produce renal responses. Subgroup analysis of the ENDEAVOR study demonstrated complete renal response rates of 15.3% among patients with relapsed/refractory MM (RRMM) treated with carfilzomib and dexamethasone, with those achieving complete renal responses demonstrating longer median progression-free survival (PFS) and overall survival (OS) than patients who did not achieve renal response [12]. However, carfilzomib has also been associated with renal toxicity [13].

Ixazomib in combination with lenalidomide and dexamethasone has shown good antitumor activity in patients with RI [14], but no studies so far have reported improvements in renal function.

One of the most widely used immunomodulatory therapies, lenalidomide, is renally excreted, and so requires dose adjustment in patients with RI, but is still considered to be effective for the management of mild-to-moderate RI [6].

Pomalidomide plus dexamethasone is well tolerated in patients with MM refractory to lenalidomide and bortezomib, including those with RI [15]. Unlike lenalidomide, pomalidomide does not require dose modification in patients with RI [16]. In a phase 3 study, 32% of patients treated with pomalidomide and low-dose dexamethasone (Pd) achieved a complete renal response, defined in accordance with the IMWG recommendations [17]. Moreover, Pd had comparable effects on OS, PFS and toxicity in patients with and without moderate RI [17]. A further study in patients with moderate RI (eGFR 30 to <45 mL/min/1.73 m²) reported a sustained complete renal response in 18.2% of patients [18].

Few data exist exploring RI among patients receiving monoclonal antibody therapy. Isatuximab (Isa) is an IgG1 monoclonal antibody that targets a specific epitope on CD38, with several mechanisms of action against MM [19]. Administering Isa in combination with Pd in preclinical studies was shown to increase its antitumor activity [19], while a phase 1b study reported encouraging response rates and PFS in patients with RRMM treated with the Isa-Pd combination [20]. These benefits were confirmed in ICARIA-MM, the first randomized, phase 3, active-controlled trial to investigate the efficacy and safety of Isa-Pd in patients with RRMM and ≥2 prior lines of therapy [21, 22]. In the primary analysis, Isa-Pd showed a statistically significant and clinically meaningful improvement in PFS versus Pd (11.53 vs 6.47 months; hazard ratio [HR] 0.596; 95% confidence interval [CI], 0.436–0.814; *P* = 0.001) [22]. The patient population in ICARIA-MM reflected the real-world setting, and included patients who were heavily pretreated (median 3 [range, 2–11] prior lines of therapy), with more than 70% of patients refractory to both lenalidomide and a proteasome inhibitor (PI). In addition, patients with eGFR ≥30 mL/min/1.73 m² were eligible for inclusion and 36.2% of patients in ICARIA-MM had RI (eGFR <60 mL/min/1.73 m²) [22]. Here, we report a prespecified subgroup analysis of ICARIA-MM comparing efficacy, renal response, and safety with Isa-Pd versus Pd, and the pharmacokinetics (PK) of Isa, in patients with and without RI.

## Subjects and methods

### Study design

ICARIA-MM (NCT02990338) was a prospective, randomized, open-label, active-controlled, multicenter phase 3 study [22]. Institutional review boards and independent ethics committees of participating institutions approved the protocol. The study was conducted according to the principles of the Declaration of Helsinki and the International Conference on Harmonisation Good Clinical Practice Guidelines. All patients provided written informed consent.

### Patients

Details of the study methodology have been reported previously [22]. Briefly, eligible patients with RRMM had received ≥2 prior lines of therapy and had failed both lenalidomide and a PI given alone or in combination. Patients had progressed within 60 days of completing the previous therapy. Patients with primary refractory disease were excluded. Patients were included if they had a baseline eGFR of ≥30 mL/min/1.73 m² (moderate RI), determined using the Modification of Diet in Renal Disease (MDRD) equation [22].

### Treatment

All patients received pomalidomide 4 mg orally on days 1–21 plus dexamethasone 40 mg (20 mg in patients ≥75 years old) orally or intravenously on days 1, 8, 15, and 22 of each 28-day cycle. Patients randomized to the Isa-Pd treatment arm also received Isa 10 mg/kg administered intravenously on days 1, 8, 15, and 22 of cycle 1, then on days 1 and 15 in each subsequent cycle.

### Study endpoints and outcomes measured

#### PFS and OS

PFS was defined as the time from randomization to first documentation of progressive disease, as determined by an independent response committee, or death. Response was assessed according to IMWG criteria using central lab data for M-protein and central review of imaging [22]. OS was defined as the time from randomization to death from any cause.

#### Clinical response

Patients were assessed for clinical response at screening/baseline, on day 1 of each treatment cycle, at the end of treatment visit (30 days after the last dose of treatment), and every 30 days until disease progression. Assessments included serum and 24-h urine M-protein quantification (by local and central laboratory), quantitative immunoglobulins (local and central laboratory), and imaging when appropriate.

#### Renal response

eGFR (calculated by MDRD) was assessed at screening, within 24 h prior to study treatment administration on days 1, 8, 15, and 22 of cycle 1, and within 24 h prior to study treatment administration on day 1 of every subsequent cycle, at the end of treatment visit, and as clinically indicated. eGFR results were classified as RI (<60 mL/min/1.73 m²) or no RI (≥60 mL/min/1.73 m²); data were also examined among patients with eGFR <45 mL/min/1.73 m². Complete renal response was defined as an improvement in eGFR from <50 mL/min/1.73 m² at baseline to ≥60 mL/min/1.73 m² in at least one post-baseline assessment (IMWG recommendations [6]). Responses were considered durable (sustained) when lasting ≥60 days. A minor renal response was defined as an improvement in eGFR from <15 mL/min/1.73 m² at baseline to ≥15–<30 mL/min/1.73 m² or from ≥15–<30 mL/min/1.73 m² at baseline to ≥30 mL/min/1.73 min² in at least one assessment during treatment.

#### Safety

Safety assessments (including adverse events, serious adverse events, laboratory parameters, vital signs, and physical examination) were performed throughout the study. All adverse events were graded according to National Cancer Institute Common Terminology for Adverse Events v4.03. Treatment-emergent adverse events (TEAEs) were those that developed or worsened from the time of signed informed consent to 30 days after last administration of study treatment.

#### PK analyses

Isa concentration data were used for population PK analysis after pooling data from the Isa-Pd arm (*N* = 148) with data from single-agent studies (*N* = 284) and an Isa-Pd phase 1b study (*N* = 44).

The PK analysis used Monolix 2018R1 software (Lixoft, Antony, France) to characterize Isa nonlinear (target-specific clearance) and linear (target-nonspecific clearance) PK and its time-dependency, and identify the source of intrinsic/extrinsic PK variability, including the effect of renal function impairment.

#### Statistical analyses

Response rates were compared between treatment groups using the stratified Cochran–Mantel–Haenszel test. PFS and OS were analyzed between treatment groups using Kaplan–Meier methodology and reported as median values with 95% CIs. HRs were estimated using the stratified Cox proportional hazards model and reported with 95% CIs. Treatment arms were compared using a one-sided log-rank test stratified by number of prior lines of therapy and age. Comparisons between patients with and without RI were observational only, with no formal statistical analysis performed. SAS 9.4 (SAS, Cary, NC) was used for all analyses.

## Results

### Patients

Altogether, 307 patients were randomized to Isa-Pd (*n* = 154) or Pd (*n* = 153). Of those, 142 and 145 patients had evaluable eGFR, respectively; 12 and 8 patients were not evaluable due to local legal restrictions on collecting race information. Of patients evaluable for MDRD, 55 (38.7%) and 49 (33.8%) had RI at baseline, of whom 19 (13.4%) and 16 (11.0%) had eGFR ≥30–<45 mL/min/1.73 m². Each arm had one patient with eGFR <30 mL/min/1.73 m² who was included in the analysis. Baseline demographics and clinical characteristics were similar between treatment arms, and between patients with and without RI at baseline with the exception that patients with RI tended to be older, have more International Staging System stage III disease and more light chain disease than patients without RI (Table 1). Patient flow has been described previously [22].

**Table 1:** Baseline demographics and clinical characteristics.

	eGFR <60 mL/min/1.73 m^2^		eGFR ≥60 mL/min/1.73 m^2^	
	Isa-Pd (*n* = 55)	Pd (*n* = 49)	Isa-Pd (*n* = 87)	Pd (*n* = 96)
Median age, years (range)	71 (39–83)	67 (41–86)	66 (36–82)	64 (41–81)
Female, *n* (%)	22 (40.0)	26 (53.1)	38 (43.7)	54 (56.3)
Age categories, *n* (%)				
<65 years	15 (27.3)	18 (36.7)	34 (39.1)	49 (51.0)
65–74 years	21 (38.2)	16 (32.7)	42 (48.3)	35 (36.5)
≥75 years	19 (34.5)	15 (30.6)	11 (12.6)	12 (12.5)
Median time since diagnosis, years (range)	4.4 (1.3–11.1)	4.5 (1.2–15.8)	4.9 (0.6–18.4)	4.0 (0.5–20.5)
Type of myeloma at diagnosis, *n* (%)				
IgA	16 (29.1)	9 (18.4)	16 (18.4)	30 (31.3)
IgG	30 (54.5)	34 (69.4)	63 (72.4)	60 (62.5)
Light chain (κ+λ)	7 (12.7)	6 (12.2)	7 (8.0)	5 (5.2)
ISS stage^a^ at diagnosis, *n* (%)				
I	7 (12.7)	9 (18.4)	27 (31.0)	29 (30.2)
II	14 (25.5)	15 (30.6)	30 (34.5)	30 (31.3)
III	23 (41.8)	19 (38.8)	16 (18.4)	24 (25.0)
ISS stage^a^ at study entry, *n* (%)				
I	14 (25.5)	7 (14.3)	45 (51.7)	42 (43.8)
II	16 (29.1)	16 (32.7)	32 (36.8)	36 (37.5)
III	25 (45.5)	25 (51.0)	7 (8.0)	16 (16.7)
Median bone marrow plasma cells at study entry, % (range)	32.0 (0–100)	30.0 (1.0–93.0)	23.6 (0–100)	30.0 (0–90.0)
Cytogenetic risk at study entry^b^, *n* (%)				
High	9 (16.4)	11 (22.4)	11 (12.6)	22 (22.9)
Standard	36 (65.5)	29 (59.2)	63 (72.4)	47 (49.0)
Missing	10 (18.2)	9 (18.4)	13 (14.9)	27 (28.1)
Median prior lines of therapy (range)	3 (2–11)	3 (2–10)	3 (2–11)	3 (2–9)
Prior therapy, *n* (%)				
Alkylating agent	49 (89.1)	47 (95.9)	80 (92.0)	93 (96.9)
Proteasome inhibitor	55 (100)	49 (100)	87 (100)	96 (100)
Lenalidomide	55 (100)	49 (100)	87 (100)	96 (100)
Refractory status, *n* (%)				
IMiD refractory	52 (94.5)	44 (89.8)	83 (95.4)	92 (95.8)
Lenalidomide refractory	51 (92.7)	42 (85.7)	81 (93.1)	90 (93.8)
PI refractory	41 (74.5)	42 (85.7)	70 (80.5)	69 (71.9)
Lenalidomide and PI refractory	39 (70.9)	37 (75.5)	65 (74.7)	66 (68.8)
Lenalidomide last line	35 (63.6)	22 (44.9)	48 (55.2)	59 (61.5)

*eGFR* estimated glomerular filtration rate, *Ig* immunoglobulin, *Isa* isatuximab, *ISS* International Staging System, *IMiD* immunomodulatory drug, *Pd* pomalidomide and dexamethasone, *PI* proteasome inhibitor.

^a^ISS staging was derived based on the combination of serum β_2_-microglobulin and albumin.

^b^High risk was defined as del(17p), t(4;14), or t(14;16) by fluorescence in situ hybridization. Cytogenetics was performed by a central laboratory with a cut-off of 50% for del(17p), and 30% for t(4;14) and t(14;16).

### Efficacy

#### PFS

**Fig. 1:**
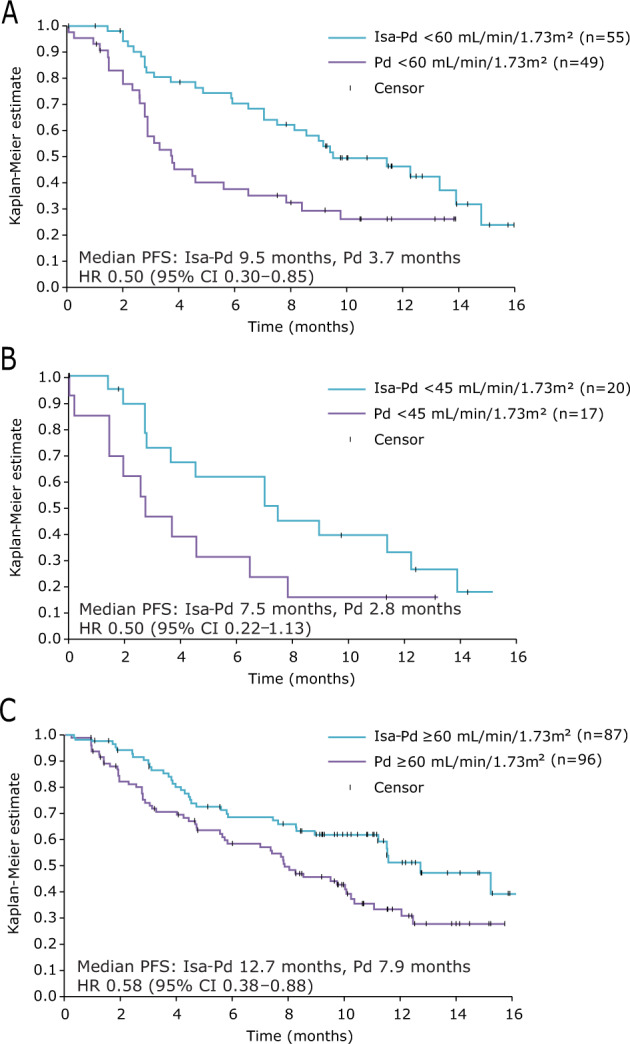
Progression-free survival. Patients with eGFR <60 mL/min/1.73 m² (**a**), <45 mL/min/1.73 m² (**b**), and ≥60 mL/min/1.73 m² (**c**) in the Isa-Pd and Pd arms. *CI* confidence interval, *eGFR* estimated glomerular filtration rate, *HR* hazard ratio, *Isa-Pd* isatuximab, pomalidomide, and dexamethasone, *Pd* pomalidomide and dexamethasone, *PFS* progression-free survival.

The PFS benefit of Isa-Pd versus Pd in patients with and without RI was consistent with that seen for the overall study population. For patients with RI, median PFS was 9.5 months with Isa-Pd (*n* = 55) and 3.7 months with Pd (*n* = 49; HR 0.50; 95% CI, 0.30–0.85) (Fig. 1a). For patients with eGFR <45 mL/min/1.73 m², median PFS was 7.5 and 2.8 months (HR 0.50; 95% CI, 0.22–1.13) for Isa-Pd (*n* = 20) and Pd (*n* = 17), respectively (Fig. 1b). In patients without RI, median PFS was 12.7 months with Isa-Pd (*n* = 87) and 7.9 months with Pd (*n* = 96; HR 0.58; 95% CI, 0.38–0.88) (Fig. 1c).

#### OS

**Fig. 2:**
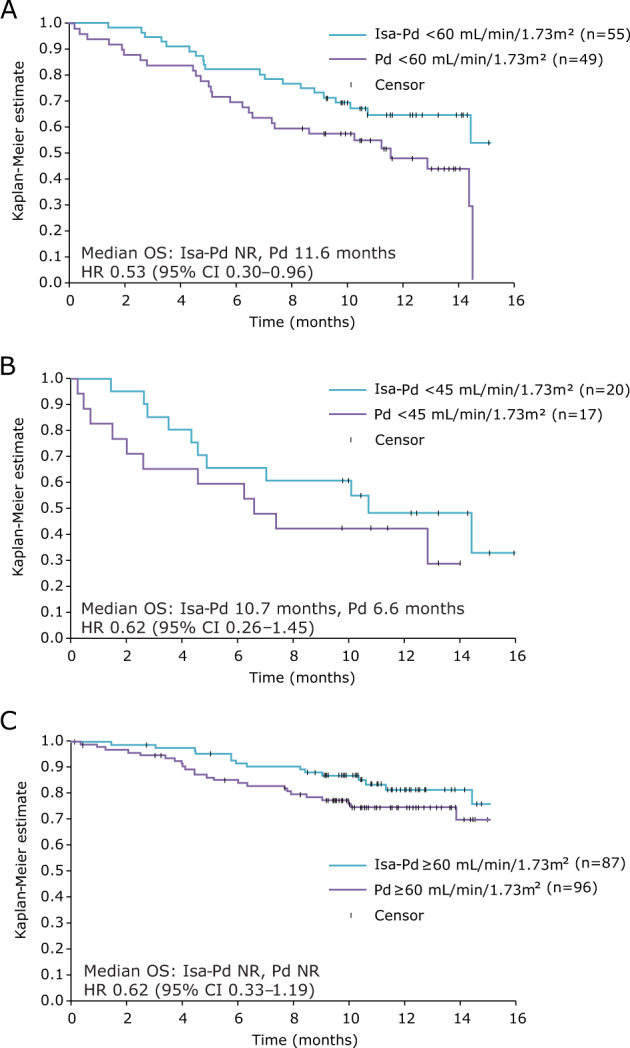
Overall survival. Patients with eGFR <60 mL/min/1.73 m² (**a**), <45 mL/min/1.73 m² (**b**) and ≥60 mL/min/1.73 m² (**c**) in the Isa-Pd and Pd arms. *CI* confidence interval, *eGFR* estimated glomerular filtration rate, *HR* hazard ratio, *Isa-Pd* isatuximab, pomalidomide, and dexamethasone, *OS* overall survival, *Pd* pomalidomide and dexamethasone, *NR* not reached.

OS data for the entire ICARIA-MM study are not yet mature, but can be analyzed in the smaller RI subpopulation. Median OS for patients with RI was not reached in the Isa-Pd arm compared with 11.6 months in the Pd arm (HR 0.53; 95% CI, 0.30–0.96; Fig. 2a). For patients with eGFR <45 mL/min/1.73 m², median OS was 10.7 versus 6.6 months (HR 0.62; 95% CI, 0.26–1.45) for Isa-Pd versus Pd (Fig. 2b). In patients without RI, median OS was not reached in either arm (HR 0.62; 95% CI, 0.33–1.19; Fig. 2c).

#### ORR

**Fig. 3:**
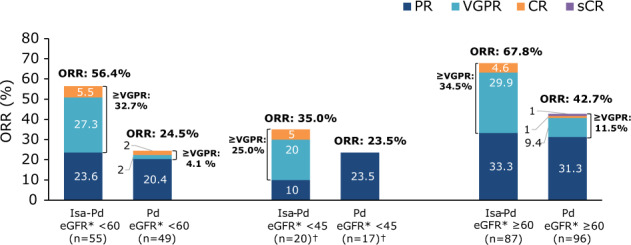
Response rates. Overall response rate and depth of response according to renal function in patients treated with Isa-Pd and Pd. ^†^One patient in each arm had eGFR <30 mL/min/1.73 m²; the treatment response was stable disease for the patient in the Isa-Pd arm and progressive disease for the patient in the Pd arm. *CR* complete response, *eGFR* estimated glomerular filtration rate, *Isa-Pd* isatuximab, pomalidomide, and dexamethasone, *ORR* overall response rate, *Pd* pomalidomide and dexamethasone, *PR* partial response, *sCR* stringent complete response, *VGPR* very good partial response.

The ORR was higher with Isa-Pd versus Pd, regardless of RI status (Fig. 3). ORR was 56.4% and 24.5% with Isa-Pd and Pd, respectively for patients with RI (odds ratio [OR] 3.98; 95% CI, 1.60–10.17). Among patients with RI, 32.7% and 4.1% had a very good partial response or better with Isa-Pd and Pd, respectively. Eight patients in the Isa-Pd arm achieved minimal residual disease negativity (sensitivity level 10^−5^), of whom three had an eGFR <60 mL/min/1.73 m². No patients in the Pd arm achieved minimal residual disease negativity. The ORR for patients with eGFR <45 mL/min/1.73 m² was 35.0% with Isa-Pd and 23.5% with Pd (OR 1.75; 95% CI, 0.34–10.11). For patients without RI, ORR was 67.8% and 42.7% with Isa-Pd and Pd, respectively (OR 2.83; 95% CI, 1.48–5.42). ORR was 68.6% for patients with eGFR ≥45–<60 mL/min/1.73 m² in the Isa-Pd arm (*n* = 35), which was noted to be similar to that of patients without RI, whereas the ORR was 25.0% for patients with mild RI in the Pd arm (*n* = 32).

#### Renal response

**Fig. 4:**
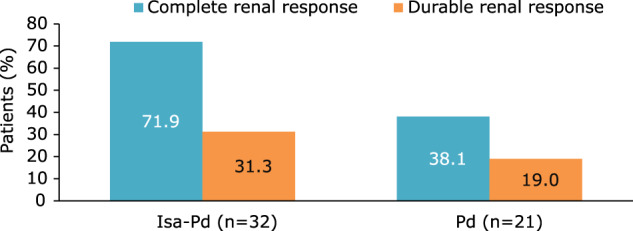
Renal response. Complete and durable (≥60 days) renal responses in patients with eGFR <50 mL/min/1.73 m² at baseline in the Isa-Pd and Pd arms. *eGFR* estimated glomerular filtration rate, *Isa-Pd* isatuximab, pomalidomide, and dexamethasone, *Pd* pomalidomide and dexamethasone.

Complete renal response occurred in 71.9% (23/32) of patients in the Isa-Pd arm and 38.1% (8/21) of those in the Pd arm (OR 4.15; 95% CI, 1.12–15.78; Fig. 4). Durable complete renal response was achieved more frequently with Isa-Pd (31.3% [10/32]) than Pd (19.0% [4/21]) with OR 1.93; 95% CI, 0.45–9.82 (Fig. 4). In addition, one patient in the Isa-Pd arm had a minor renal response. Median time to renal response was 3.4 and 7.3 weeks for Isa-Pd and Pd, respectively.

In patients evaluable for renal response (baseline eGFR <50 mL/min/1.73 m²) in both the Isa-Pd and and Pd arms, tumor response rates were higher in patients with renal response than patients without renal response (Isa-Pd 52% vs 44%; Pd 38% vs 23%).

Fewer patients in the Isa-Pd arm progressed to end-stage renal disease (ESRD; eGFR <15 mL/min/1.73 m²) during treatment with Isa-Pd versus Pd (2.9% vs 7.9%). Among patients with moderate RI at baseline, renal function worsened to severe RI or ESRD in 22.6% (12/53) of patients in the Isa-Pd arm and 34.8% (16/46) of patients in the Pd arm (OR 0.55; 95% CI, 0.20–1.45).

### Safety

#### Treatment exposure

The median (range) number of cycles for patients with and without RI was 10 (1–18) and 10 (1–19) cycles with Isa-Pd (54 and 86 treated patients), and 5 (1–16) and 7 (1–18) cycles with Pd (47 and 94 treated patients), respectively. The median duration of exposure for patients with and without RI was 41.6 (4.0–74.1) and 43.6 (3.1–76.7) weeks with Isa-Pd, and 19.3 (1.0–65.0) and 28.6 (1.7–73.7) weeks with Pd, respectively.

#### Adverse events

All patients with RI experienced TEAEs, while 85 (98.8%) and 91 (96.8%) patients without RI experienced TEAEs in the Isa-Pd and Pd arms, respectively (Table 2). The incidence of grade 5 TEAEs, serious TEAEs, and TEAEs leading to definitive treatment discontinuation was higher in patients with versus without RI for both treatment arms (Table 2). When all grade TEAEs were analyzed by type, there was a ≥10% higher incidence of cardiac disorders and infections in patients with RI versus without RI in the Isa-Pd arm. The incidence of grade ≥3 TEAEs was similar for patients with versus without RI (90.7% vs 86%) in the Isa-Pd arm (Table 2). By type, in patients with RI, there was a ≥10% higher incidence of grade ≥3 musculoskeletal disorders and infections in the Isa-Pd arm. Among the grade ≥3 infections, there was a ≥10% higher incidence of pneumonia that was also seen in the Pd arm (Table 2).

**Table 2:** TEAEs by baseline renal function and treatment arm.

*n* (%)	eGFR <60 mL/min/1.73 m²		eGFR ≥60 mL/min/1.73 m²	
	Isa-Pd (*n* = 54)	Pd (*n* = 47)	Isa-Pd (*n* = 86)	Pd (*n* = 94)
Median treatment duration, weeks (range)	41.6 (4.0–74.1)	19.3 (1.0–65.0)	43.6 (3.1–76.7)	28.6 (1.7–73.7)
Any TEAE	54 (100.0)	47 (100.0)	85 (98.8)	91 (96.8)
Infections^a^	49 (90.7)	30 (63.8)	67 (77.9)	62 (66.0)
Cardiac disorders^a^	12 (22.2)	1 (2.1)	10 (11.6)	5 (5.3)
Gastrointestinal disorders^a^	30 (55.6)	28 (59.6)	46 (53.5)	44 (46.8)
General disorders and administration site conditions^a^	29 (53.7)	32 (68.1)	45 (52.3)	53 (56.4)
Grade ≥3 TEAE	49 (90.7)	37 (78.7)	74 (86.0)	63 (67.0)
Infections^a^	30 (55.6)	18 (38.3)	33 (38.4)	27 (28.7)
Pneumonia^b^	14 (25.9)	11 (23.4)	11 (12.8)	12 (12.8)
Musculoskeletal disorders^a^	9 (16.7)	3 (6.4)	3 (3.5)	3 (3.2)
Grade 5 TEAE	5 (9.3)	6 (12.8)	3 (3.5)	6 (6.4)
Serious TEAE	42 (77.8)	28 (59.6)	44 (51.2)	48 (51.1)
TEAE leading to definitive treatment discontinuation	6 (11.1)	7 (14.9)	5 (5.8)	11 (11.7)

*eGFR* estimated glomerular filtration rate, *Isa* isatuximab, *Pd* pomalidomide and dexamethasone, *RI* renal impairment, *SOC* system organ class, *TEAE* treatment-emergent adverse event.

^a^SOC with TEAEs with an incidence ≥10% greater in patients with versus without RI in the same arm.

^b^Grade ≥3 TEAE with an incidence ≥10% greater in patients with versus without RI in the same arm, among SOCs defined in^a^.

Table 3 shows the most common TEAEs according to treatment arm and baseline renal function. The most common TEAEs (≥15 % in all arms) were neutropenia, upper respiratory tract infections, pneumonia, diarrhea, constipation, fatigue, back pain, and asthenia. Infections were most frequently respiratory infections and less frequently urinary tract infections. The incidence of upper respiratory tract infections appeared similar in patients with and without RI but were observed more frequently with Isa-Pd than Pd, while lower respiratory infections were observed more frequently in patient with RI in both treatment arms. The types of infection experienced by patients in the Isa-Pd and Pd arms stratified by renal function are shown in Supplementary Table. Notably, there was a similar exposure-adjusted rate of serious infections in the overall population of the two study arms (0.72 per patient year in the Isa-Pd arm and 0.67 per patient-year in the Pd arm). Infusion-related reactions were observed in around one-third of patients treated with Isa-Pd in both the RI and non-RI groups.

**Table 3:** Most common (occurring in ≥15% of patients) TEAEs by baseline renal function and treatment arm.

*n* (%)	eGFR <60 mL/min/1.73 m^2^		eGFR ≥60 mL/min/1.73 m^2^	
	Isa-Pd (*n* = 54)	Pd (*n* = 47)	Isa-Pd (*n* = 86)	Pd (*n* = 94)
Any	54 (100.0)	47 (100.0)	85 (98.8)	91 (96.8)
Infections				
Upper respiratory tract infection	16 (29.6)	6 (12.8)	27 (31.4)	19 (20.2)
Bronchitis	11 (20.4)	2 (4.3)	21 (24.4)	9 (9.6)
Pneumonia	16 (29.6)	12 (25.5)	15 (17.4)	14 (14.9)
Blood and lymphatic system disorders				
Neutropenia	24 (44.4)	19 (40.4)	43 (50.0)	29 (30.9)
Thrombocytopenia	8 (14.8)	8 (17.0)	10 (11.6)	9 (9.6)
Febrile neutropenia	9 (16.7)	3 (6.4)	8 (9.3)	0
Respiratory, thoracic and mediastinal disorders				
Dyspnea	5 (9.3)	5 (10.6)	16 (18.6)	9 (9.6)
Gastrointestinal disorders				
Diarrhea	15 (27.8)	14 (29.8)	22 (25.6)	14 (14.9)
Constipation	7 (13.0)	8 (17.0)	16 (18.6)	17 (18.1)
Nausea	6 (11.1)	7 (14.9)	16 (18.6)	7 (7.4)
Musculoskeletal and connective tissue disorders				
Back pain	14 (25.9)	7 (14.9)	11 (12.8)	15 (16.0)
General disorders and administration site conditions				
Fatigue	10 (18.5)	13 (27.7)	16 (18.6)	18 (19.1)
Peripheral edema	5 (9.3)	6 (12.8)	15 (17.4)	10 (10.6)
Pyrexia	12 (22.2)	10 (21.3)	8 (9.3)	9 (9.6)
Asthenia	6 (11.1)	11 (23.4)	12 (14.0)	15 (16.0)
Procedural complications				
Infusion-related reaction	18 (33.3)	1 (2.1)	34 (39.5)	1 (1.1)

*eGFR* estimated glomerular filtration rate, *Isa* isatuximab, *Pd* pomalidomide and dexamethasone, *TEAE* treatment-emergent adverse event.

In patients with RI at baseline, the incidence of grade ≥3 and serious TEAEs was higher with Isa-Pd (90.7% and 77.8%) than with Pd (78.7% and 59.6%; Table 2). When adjusted for difference in exposure, the event rate of serious TEAEs per patient-year was 1.90 with Isa-Pd and 1.94 with Pd. In patients without RI at baseline, the incidence of grade ≥3 and serious TEAEs was 86.0% and 51.2% versus 67.0% and 51.1% in the Isa-Pd and Pd groups, respectively. There was no increased incidence of grade 5 TEAEs or of TEAEs leading to treatment discontinuation with Isa-Pd versus Pd in patients with RI (9.3% vs 12.8% and 11.1% vs 14.9%) and in patients without RI (3.5% vs 6.4% and 5.8% vs 11.7%; Table 2).

### PK analyses in patients with RI at baseline

Renal function was not identified as an influential covariate on Isa PK. The model predicted a mean area under the curve over the dosing interval of 2 weeks at steady state of 73,200 µg.h/mL (coefficient of variation [CV] 75.6%) in patients with severe RI (*N* = 12; eGFR <30 mL/min/1.73 m²), of 74,600 µg.h/mL (CV 59.2%) in patients with moderate RI (*N* = 163; eGFR ≥30 and <60 mL/min/1.73 m²), and of 77,700 µg.h/mL (CV 58.0%) in patients with mild RI (*N* = 192; eGFR ≥60 mL/min/1.73 m² and <90 mL/min/1.73 m²). Taking account of interpatient variability, these values are comparable to those of patients with normal renal function (78,400 µg.h/mL; CV 53.6%).

## Discussion

Renal impairment is an independent predictor of poor prognosis in patients with RRMM, and there is a critical need for anti-myeloma therapies that also improve renal function. This prespecified subgroup analysis of ICARIA-MM—the first randomized phase 3 study to demonstrate a significant survival benefit of an anti-CD38 therapy (Isa) plus Pd versus Pd in heavily pretreated patients with RRMM [22]—shows that Isa-Pd is also efficacious with a manageable safety profile in patients with RI.

The addition of Isa to the Pd backbone in patients with RI in ICARIA-MM resulted in a HR for PFS of 0.50 (95% CI, 0.30–0.85) in favor of patients receiving Isa-Pd versus Pd, consistent with the observed PFS benefit of Isa-Pd in patients without RI (HR 0.58; 95% CI, 0.38–0.88) and the overall study population (HR 0.596; 95% CI, 0.44–0.81) [22]. PFS observed among patients receiving Pd in ICARIA-MM (3.7 months) was also consistent with previously recorded results of the MM-003 study, where a similar median PFS of 4.0 months in patients with RI (eGFR <60 mL/min/1.73 m²) was observed for patients treated with Pd [17].

The ORR was greater with Isa-Pd than Pd in patients with RI (56.4% vs 24.5%) and without RI (67.8% vs 42.7%), again consistent with the overall study population (Isa-Pd, 60.4%; Pd, 35.3%) [22]. Indeed, the ORR with Isa-Pd was similar between patients with mild RI (eGFR ≥45–<60 mL/min/1.73 m²; ORR 68.6%) and patients without RI.

Although OS data for ICARIA-MM are not fully mature, a favorable HR of 0.53 can already be observed with the upper limit of the 95% CI not crossing 1 (95% CI, 0.30–0.96).

Notably, compared with Pd, Isa-Pd increased the proportion of RRMM patients with RI who achieved both complete (71.9% with Isa-Pd, 38.1% with Pd) and sustained (≥60 days; 31.3% vs 19.0%) renal responses, supporting a role for Isa-Pd in achieving durable reversal of RI. Correspondingly, fewer patients in the Isa-Pd group compared with the Pd group experienced worsening of renal function to severe RI or ESRD. In these renally impaired patients, tumor response rates were higher in those with renal response than those without renal response. The higher rate of renal response experienced by patients in the Isa-Pd arm contributes importantly to the higher tumor response and hence benefit of this regimen over Pd.

The results of the ICARIA-MM subgroup analysis provide the first evidence of improvement in renal function with a CD38-targeted therapy in patients with RRMM. Data on the anti-CD38 agent daratumumab in RRMM patients with RI are currently limited to small uncontrolled studies or case reports with single patients [23, 24]. Data on elotuzumab, a humanized monoclonal antibody that binds signaling lymphocyte activation molecule F7 (SLAMF7), are also lacking in patients with RRMM and RI [5]. In a phase 1b study of elotuzumab in combination with lenalidomide and dexamethasone, there were no complete renal responses observed; two patients with severe RI showed minor renal responses. ORRs observed among patients with severe RI and ESRD were 67 and 56%, respectively, compared with an ORR of 75% among patients with normal renal function [25]. The phase 2 ELOQUENT-3 study of elotuzumab in combination with Pd in patients with RRMM excluded patients with creatinine clearance <45 mL/min, hence there are no data on efficacy or safety data for elotuzumab in an RI population below this threshold [5].

In contrast with lenalidomide [6], pomalidomide can be safely administered to patients with RI without dose modification [26], and has previously been investigated in subgroups of patients with RI. Similar renal responses to the Pd arm of ICARIA-MM were observed in a subgroup analysis of the phase 3 MM-003 trial comparing Pd versus high-dose dexamethasone in RRMM [17]. The complete renal response rate among patients with RI was 42% with Pd in MM-003, consistent with 38.1% for patients receiving Pd in ICARIA-MM. Pomalidomide in combination with low-dose dexamethasone was also investigated in the phase 2 MM-013 trial. Among patients with moderate RI (eGFR 30 to <45 mL/min/1.73 m²), sustained renal responses were observed in 18.2% of patients [18], mirroring the 19.0% durable complete renal response rate in the Pd arm of ICARIA-MM in patients with creatinine clearance <50 mL/min/1.73 m^2^.

The addition of Isa to the Pd backbone had a manageable safety profile in RRMM patients with and without RI. The incidence of TEAEs of any grade or of grade ≥3 was broadly similar between the renal subgroups. TEAEs observed to occur at a more than 10% higher incidence in the Isa-Pd arm in patients with versus without RI were cardiac disorders (any grade), musculoskeletal disorders (grade ≥3), and infections (any grade and grade ≥3), with pneumonia identified as a driver behind the increased grade ≥3 infections. Grade ≥3 pneumonia was also observed to have a more than 10% higher incidence in patients with RI than those without RI in the Pd arm, suggesting that patients with RI should be carefully monitored for lower respiratory tract infections. Incidence of infusion-related reactions were similar between the RI and non-RI groups. Among patients with RI at baseline, the incidence of grade ≥3 and serious TEAEs was greater in the Isa-Pd versus Pd arm, but this did not translate to an increased incidence of grade 5 TEAEs or TEAEs leading to treatment discontinuation. Notably, patients in the Isa-Pd arm had increased treatment exposure compared with patients in the Pd arm, which may have led to a higher incidence of AEs in the Isa-Pd arm. When adjusted for difference in exposure, the event rate of serious TEAEs per patient year for patients with RI was similar in the Isa-Pd and Pd arms, and the event rate of serious infections per patient-year was similar in the two study arms.

Rather than being renally excreted, protein therapeutics are eliminated from the body nearly exclusively by proteolysis, to which the kidneys may substantially contribute as a major site for protein catabolism [27]. The PK of Isa was not modified by altered renal function, which was as expected since monoclonal antibodies are not renally excreted. In addition, it has been shown that the PK of pomalidomide is not modified in patients with RI [28]. Therefore, there is no PK-based need for dose adjustment in RI patients.

The large study population, combined with the multicenter, prospective design, support the study findings as representative of Isa safety and efficacy in these patients. Furthermore, fixed-volume infusion (250 mL) of Isa may help with the management of fluid balance in patients with RI [6, 29]. While the study used the validated IMWG criteria to classify RI status, this was not a stratification factor in the ICARIA-MM trial, which likely explains small imbalances between the renal function subgroups. We also acknowledge that few patients in this study had severe RI and none were receiving hemodialysis; therefore, our analysis is most applicable to the moderate RI population.

In summary, the addition of Isa to Pd improved PFS and ORR in patients with RI, consistent with the benefit observed in patients without RI and the overall RRMM study population. Isa-Pd also increased the number of patients with reversal of RI and sustained renal responses compared with Pd. Based on these findings, and the absence of any need for dose adjustments based on renal function, Isa-Pd represents a valuable treatment option for patients with RRMM presenting with renal dysfunction.

## Supplementary information

Supplementary Table

## References

[1] Clark AD, Shetty A, Soutar R (1999). Renal failure and multiple myeloma: pathogenesis and treatment of renal failure and management of underlying myeloma. Blood Rev..

[2] Eleutherakis-Papaiakovou V, Bamias A, Gika D, Simeonidis A, Pouli A, Anagnostopoulos A (2007). Renal failure in multiple myeloma: incidence, correlations, and prognostic significance. Leuk Lymphoma..

[3] San-Miguel JF, Richardson PG, Sonneveld P, Schuster MW, Irwin D, Stadtmauer EA (2008). Efficacy and safety of bortezomib in patients with renal impairment: results from the APEX phase 3 study. Leukemia..

[4] San-Miguel JF, Hungria VT, Yoon SS, Beksac M, Dimopoulos MA, Elghandour A (2014). Panobinostat plus bortezomib and dexamethasone versus placebo plus bortezomib and dexamethasone in patients with relapsed or relapsed and refractory multiple myeloma: a multicentre, randomised, double-blind phase 3 trial. Lancet Oncol.

[5] Dimopoulos MA, Dytfeld D, Grosicki S, Moreau P, Takezako N, Hori M (2018). Elotuzumab plus pomalidomide and dexamethasone for multiple myeloma. N Engl J Med..

[6] Dimopoulos MA, Sonneveld P, Leung N, Merlini G, Ludwig H, Kastritis E (2016). International Myeloma Working Group recommendations for the diagnosis and management of myeloma-related renal impairment. J Clin Oncol..

[7] Dimopoulos MA, Terpos E, Chanan-Khan A, Leung N, Ludwig H, Jagannath S (2010). Renal impairment in patients with multiple myeloma: a consensus statement on behalf of the International Myeloma Working Group. J Clin Oncol..

[8] Dimopoulos MA, Roussou M, Gavriatopoulou M, Psimenou E, Eleutherakis-Papaiakovou E, Migkou M (2016). Bortezomib-based triplets are associated with a high probability of dialysis independence and rapid renal recovery in newly diagnosed myeloma patients with severe renal failure or those requiring dialysis. Am J Hematol.

[9] Dimopoulos MA, Richardson PG, Schlag R, Khuageva NK, Shpilberg O, Kastritis E (2009). VMP (Bortezomib, Melphalan, and Prednisone) is active and well tolerated in newly diagnosed patients with multiple myeloma with moderately impaired renal function, and results in reversal of renal impairment: cohort analysis of the phase III VISTA study. J Clin Oncol..

[10] Scheid C, Sonneveld P, Schmidt-Wolf IG, van der Holt B, el Jarari L, Bertsch U (2014). Bortezomib before and after autologous stem cell transplantation overcomes the negative prognostic impact of renal impairment in newly diagnosed multiple myeloma: a subgroup analysis from the HOVON-65/GMMG-HD4 trial. Haematologica..

[11] Moreau P, Pylypenko H, Grosicki S, Karamanesht I, Leleu X, Rekhtman G (2015). Subcutaneous versus intravenous bortezomib in patients with relapsed multiple myeloma: subanalysis of patients with renal impairment in the phase III MMY-3021 study. Haematologica..

[12] Dimopoulos M, Siegel D, White DJ, Boccia R, Iskander KS, Yang Z (2019). Carfilzomib vs bortezomib in patients with multiple myeloma and renal failure: a subgroup analysis of ENDEAVOR. Blood..

[13] Dimopoulos MA, Roussou M, Gavriatopoulou M, Psimenou E, Ziogas D, Eleutherakis-Papaiakovou E (2017). Cardiac and renal complications of carfilzomib in patients with multiple myeloma. Blood Adv.

[14] Moreau P, Masszi T, Grzasko N, Bahlis NJ, Hansson M, Pour L (2016). Oral ixazomib, lenalidomide, and dexamethasone for multiple myeloma. N Engl J Med..

[15] Maciocia N, Melville A, Cheesman S, Sharpley F, Ramasamy K, Streetly M (2017). Real-world use of pomalidomide and dexamethasone in double refractory multiple myeloma suggests benefit in renal impairment and adverse genetics: a multi-centre UK experience. Br J Haematol..

[16] Dimopoulos MA, Leleu X, Palumbo A, Moreau P, Delforge M, Cavo M (2014). Expert panel consensus statement on the optimal use of pomalidomide in relapsed and refractory multiple myeloma. Leukemia..

[17] Weisel KC, Dimopoulos MA, Moreau P, Lacy MQ, Song KW, Delforge M (2016). Analysis of renal impairment in MM-003, a phase III study of pomalidomide + low - dose dexamethasone versus high - dose dexamethasone in refractory or relapsed and refractory multiple myeloma. Haematologica..

[18] Dimopoulos M, Weisel K, van de Donk N, Ramasamy K, Gamberi B, Streetly M (2018). Pomalidomide plus low-dose dexamethasone in patients with relapsed/refractory multiple myeloma and renal impairment: Results from a Phase II trial. J Clin Oncol.

[19] Jiang H, Acharya C, An G, Zhong M, Feng X, Wang L (2016). SAR650984 directly induces multiple myeloma cell death via lysosomal-associated and apoptotic pathways, which is further enhanced by pomalidomide. Leukemia..

[20] Mikhael J, Richardson P, Usmani SZ, Raje N, Bensinger W, Karanes C (2019). A phase 1b study of isatuximab plus pomalidomide/dexamethasone in relapsed/refractory multiple myeloma. Blood..

[21] Richardson PG, Attal M, Rajkumar SV, San Miguel J, Beksac M, Spicka I (2019). A phase III randomized, open label, multicenter study comparing isatuximab, pomalidomide, and low-dose dexamethasone versus pomalidomide and low-dose dexamethasone in patients with relapsed/refractory multiple myeloma (RRMM). J Clin Oncol.

[22] Attal M, Richardson PG, Rajkumar SV, San-Miguel J, Beksac M, Spicka I (2019). Isatuximab plus pomalidomide and low-dose dexamethasone versus pomalidomide and low-dose dexamethasone in patients with relapsed and refractory multiple myeloma (ICARIA-MM): a randomised, multicentre, open-label, phase 3 study. Lancet..

[23] Cejalvo MJ, Legarda M, Abella E, Cabezudo E, Encinas C, García-Feria A (2017). Activity and safety of daratumumab monotherapy in patients with relapsed and refractory multiple myeloma requiring dialysis: Preliminary results of Spanish, retrospective, multicenter trial. Blood..

[24] Rocchi S, Tacchetti P, Pantani L, Mancuso K, Zannetti B, Cavo M (2018). Safety and efficacy of daratumumab in dialysis-dependent renal failure secondary to multiple myeloma. Haematologica..

[25] Berdeja J, Jagannath S, Zonder J, Badros A, Kaufman JL, Manges R (2016). Pharmacokinetics and safety of elotuzumab combined with lenalidomide and dexamethasone in patients with multiple myeloma and various levels of renal impairment: Results of a Phase Ib study. Clin Lymphoma Myeloma Leuk..

[26] Usami E, Kimura M, Takenaka S, Iwai M, Teramachi H, Yoshimura T (2019). Tolerability and safety of real-world use of pomalidomide in patients with relapsed/refractory multiple myeloma. Mol Clin Oncol..

[27] Meibohm B, Zhou H (2012). Characterizing the impact of renal impairment on the clinical pharmacology of biologics. J Clin Pharmacol.

[28] Li Y, Wang X, O’Mara E, Dimopoulos MA, Sonneveld P, Weisel KC (2017). Population pharmacokinetics of pomalidomide in patients with relapsed or refractory multiple myeloma with various degrees of impaired renal function. Clin Pharmacol..

[29] Usmani S, Karanes C, Bensinger W, D’Souza A, Raje N, Tuchman S (2019). Preliminary data: Phase 1b study of feasibility/safety of isatuximab short duration fixed volume infusion in combination with pomalidomide and dexamethasone for relapsed/refractory multiple myeloma: Ps1390. HemaSphere.

