# Supplementary material for: Isatuximab plus pomalidomide and dexamethasone in relapsed/refractory multiple myeloma patients with renal impairment: ICARIA-MM subgroup analysis
Source: Leukemia. 2020 May 23;35(2):562–72. doi: 10.1038/s41375-020-0868-z (PMC7862055; doi:10.1038/s41375-020-0868-z)
Supplement: Supplementary file 1 — Supplementary Table [file 41375_2020_868_MOESM1_ESM.docx]

**Supplementary Table**. Infection TEAEs by baseline renal function and treatment arm.

| *n* (%) | **eGFR <60 mL/min/1.73m^2^** | | **eGFR ≥60 mL/min/1.73m^2^** | |  |
| --- | --- | --- | --- | --- | --- |
|  | **Isa-Pd (*n*=54)** | **Pd (*n*=47)** | **Isa-Pd (*n*=86)** | **Pd (*n*=94)** | |
| By etiology |  |  |  |  | |
| Bacterial | 8 (14.8) | 3 (6.4) | 8 (9.3) | 4 (4.3) | |
| Viral | 15 (27.8) | 9 (19.1) | 18 (20.9) | 11 (11.7) | |
| Unspecified | 43 (79.6) | 27 (57.4) | 64 (74.4) | 57 (60.6) | |
| *Upper and lower respiratory tract infections, by preferred term* | | | | | |
| Respiratory tract infection | 3 (5.6) | 3 (6.4) | 3 (3.5) | 3 (3.2) | |
| Respiratory tract infection viral | 2 (3.7) | 0 | 1 (1.2) | 2 (2.1) | |
| Upper respiratory tract infection | 16 (29.6) | 6 (12.8) | 27 (31.4) | 19 (20.2) | |
| Upper respiratory tract infection bacterial | 1 (1.9) | 0 | 0 | 0 | |
| Fungal pharyngitis | 0 | 0 | 1 (1.2) | 0 | |
| Chronic sinusitis | 0 | 1 (2.1) | 0 | 0 | |
| Laryngitis | 1 (1.9) | 1 (2.1) | 1 (1.2) | 0 | |
| Nasopharyngitis | 5 (9.3) | 1 (2.1) | 9 (10.5) | 6 (6.4) | |
| Pharyngitis | 1 (1.9) | 0 | 2 (2.3) | 1 (1.1) | |
| Rhinitis | 3 (5.6) | 0 | 2 (2.3) | 5 (5.3) | |
| Sinusitis | 0 | 1 (2.1) | 1 (1.2) | 3 (3.2) | |
| Tonsillitis | 0 | 0 | 1 (1.2) | 0 | |
| Tracheitis | 0 | 0 | 2 (2.3) | 0 | |
| Lower respiratory tract infection | 5 (9.3) | 5 (10.6) | 3 (3.5) | 3 (3.2) | |
| Bronchitis | 11 (20.4) | 2 (4.3) | 21 (24.4) | 9 (9.6) | |
| Pneumonia | 16 (29.6) | 12 (25.5) | 15 (17.4) | 14 (14.9) | |
| Bronchiolitis | 1 (1.9) | 0 | 2 (2.3) | 0 | |
| Lung infection | 2 (3.7) | 1 (2.1) | 1 (1.2) | 3 (3.2) | |
| Pneumocystis jirovecii pneumonia | 0 | 2 (4.3) | 3 (3.5) | 2 (2.1) | |
| Pneumonia viral | 1 (1.9) | 0 | 2 (2.3) | 0 | |
| Atypical pneumonia | 0 | 0 | 2 (2.3) | 0 | |
| Pneumonia bacterial | 1 (1.9) | 0 | 0 | 0 | |
| Pneumonia haemophilus | 2 (3.7) | 0 | 0 | 0 | |
| Pneumonia influenzal | 0 | 2 (4.3) | 2 (2.3) | 0 | |
| Pneumonia fungal | 0 | 0 | 1 (1.2) | 1 (1.1) | |
| Pneumonia pneumococcal | 0 | 0 | 1 (1.2) | 0 | |
| Bronchitis viral | 0 | 1 (2.1) | 0 | 1 (1.1) | |
| Bronchopulmonary aspergillosis | 1 (1.9) | 0 | 0 | 0 | |
| Candida pneumonia | 0 | 1 (2.1) | 0 | 0 | |
| Lower respiratory tract infection viral | 0 | 1 (2.1) | 0 | 0 | |
| Pneumonia streptococcal | 0 | 0 | 0 | 1 (1.1) | |
| Sputum purulent | 0 | 1 (2.1) | 0 | 0 | |
| Non-respiratory infections, by preferred term | | | | | |
| Urinary tract infection | 4 (7.4) | 7 (14.9) | 9 (10.5) | 6 (6.4) | |
| Oral herpes | 4 (7.4) | 1 (2.1) | 3 (3.5) | 2 (2.1) | |
| Influenza | 6 (11.1) | 4 (8.5) | 3 (3.5) | 4 (4.3) | |
| Cellulitis | 1 (1.9) | 1 (2.1) | 2 (2.3) | 2 (2.1) | |
| Diverticulitis | 2 (3.7) | 0 | 1 (1.2) | 2 (2.1) | |
| Herpes zoster | 2 (3.7) | 0 | 3 (3.5) | 1 (1.1) | |
| Sepsis | 2 (3.7) | 1 (2.1) | 2 (2.3) | 1 (1.1) | |
| Clostridium difficile colitis | 0 | 0 | 1 (1.2) | 0 | |
| Conjunctivitis | 0 | 0 | 1 (1.2) | 1 (1.1) | |
| Gastroenteritis | 0 | 1 (2.1) | 2 (2.3) | 1 (1.1) | |
| Skin infection | 1 (1.9) | 0 | 2 (2.3) | 0 | |
| Acarodermatitis | 1 (1.9) | 0 | 1 (1.2) | 0 | |
| Furuncle | 0 | 0 | 1 (1.2) | 0 | |
| Herpes simplex | 1 (1.9) | 0 | 1 (1.2) | 0 | |
| Otitis media | 0 | 0 | 0 | 2 (2.1) | |
| Otitis media acute | 1 (1.9) | 0 | 1 (1.2) | 0 | |
| Staphylococcal bacteraemia | 1 (1.9) | 0 | 0 | 0 | |
| Abscess limb | 0 | 0 | 1 (1.2) | 0 | |
| Adenovirus infection | 0 | 0 | 1 (1.2) | 0 | |
| Angular cheilitis | 0 | 0 | 1 (1.2) | 0 | |
| Arthritis bacterial | 1 (1.9) | 0 | 0 | 0 | |
| Clostridial infection | 0 | 0 | 1 (1.2) | 0 | |
| Clostridium colitis | 1 (1.9) | 0 | 0 | 0 | |
| Cytomegalovirus gastritis | 0 | 0 | 1 (1.2) | 0 | |
| Cytomegalovirus infection | 1 (1.9) | 0 | 0 | 0 | |
| Eye infection | 0 | 0 | 1 (1.2) | 1 (1.1) | |
| Folliculitis | 0 | 1 (2.1) | 0 | 0 | |
| Gastroenteritis enteroviral | 1 (1.9) | 0 | 0 | 0 | |
| Genital candidiasis | 0 | 0 | 1 (1.2) | 0 | |
| Haemophilus infection | 0 | 0 | 1 (1.2) | 0 | |
| Herpes zoster disseminated | 0 | 0 | 1 (1.2) | 0 | |
| Infection | 0 | 0 | 1 (1.2) | 1 (1.1) | |
| Medical device site infection | 1 (1.9) | 0 | 0 | 0 | |
| Nail infection | 0 | 0 | 1 (1.2) | 0 | |
| Oesophageal candidiasis | 0 | 0 | 1 (1.2) | 0 | |
| Oral candidiasis | 0 | 3 (6.4) | 1 (1.2) | 2 (2.1) | |
| Oral fungal infection | 0 | 0 | 0 | 1 (1.1) | |
| Orchitis | 0 | 0 | 1 (1.2) | 0 | |
| Parainfluenzae virus infection | 1 (1.9) | 0 | 0 | 0 | |
| Postoperative wound infection | 1 (1.9) | 0 | 0 | 0 | |
| Pseudomonal bacteraemia | 0 | 0 | 1 (1.2) | 0 | |
| Pseudomonas infection | 0 | 0 | 1 (1.2) | 0 | |
| Pyuria | 1 (1.9) | 0 | 0 | 0 | |
| Septic shock | 0 | 1 (2.1) | 1 (1.2) | 2 (2.1) | |
| Skin candida | 0 | 0 | 1 (1.2) | 0 | |
| Soft tissue infection | 0 | 0 | 1 (1.2) | 0 | |
| Subcutaneous abscess | 1 (1.9) | 0 | 0 | 0 | |
| Tooth infection | 1 (1.9) | 1 (2.1) | 0 | 0 | |
| Varicella | 0 | 0 | 1 (1.2) | 0 | |
| Vulvovaginal candidiasis | 0 | 0 | 1 (1.2) | 0 | |
| Anorectal infection bacterial | 0 | 1 (2.1) | 0 | 0 | |
| Body tinea | 0 | 0 | 0 | 1 (1.1) | |
| Cystitis | 0 | 0 | 0 | 3 (3.2) | |
| Cytomegalovirus gastrointestinal infection | 0 | 0 | 0 | 1 (1.1) | |
| Ear infection | 0 | 0 | 0 | 2 (2.1) | |
| Echinococciasis | 0 | 0 | 0 | 1 (1.1) | |
| Erysipelas | 0 | 1 (2.1) | 0 | 0 | |
| Escherichia sepsis | 0 | 0 | 0 | 1 (1.1) | |
| Gastrointestinal fungal infection | 0 | 1 (2.1) | 0 | 0 | |
| Genital infection fungal | 0 | 0 | 0 | 1 (1.1) | |
| Infected skin ulcer | 0 | 0 | 0 | 1 (1.1) | |
| Oropharyngeal candidiasis | 0 | 1 (2.1) | 0 | 0 | |
| Pyelonephritis | 0 | 0 | 0 | 1 (1.1) | |
| Pyelonephritis acute | 0 | 0 | 0 | 1 (1.1) | |
| Tinea pedis | 0 | 0 | 0 | 1 (1.1) | |
| Tooth abscess | 0 | 0 | 0 | 1 (1.1) | |
| Vaginal infection | 0 | 0 | 0 | 1 (1.1) | |
| Wound infection | 0 | 1 (2.1) | 0 | 0 | |

*eGFR* estimated glomerular filtration rate, *Isa* isatuximab, *Pd* pomalidomide and dexamethasone, *TEAE* treatment-emergent adverse event
